# Factors Associated With Working in Remote Indonesia: A National Cross-Sectional Study of Early-Career Doctors

**DOI:** 10.3389/fmed.2021.594695

**Published:** 2021-05-13

**Authors:** Likke Prawidya Putri, Deborah Jane Russell, Belinda Gabrielle O'Sullivan, Rebecca Kippen

**Affiliations:** ^1^Department of Health Policy and Management, Faculty of Medicine, Public Health and Nursing, Universitas Gadjah Mada, Yogyakarta, Indonesia; ^2^School of Rural Health, Faculty of Medicine, Nursing, and Health Sciences, Monash University, Bendigo, VIC, Australia; ^3^Menzies School of Health Research, Alice Springs, NT, Australia; ^4^Rural Clinical School, University of Queensland, Toowoomba, QLD, Australia

**Keywords:** rural health services, physician practice, low- and middle-income countries, health workforce maldistribution, career choice, professional practice location

## Abstract

**Background:** Doctor shortages in remote areas of Indonesia are amongst challenges to provide equitable healthcare access. Understanding factors associated with doctors' work location is essential to overcome geographic maldistribution. Focused analyses of doctors' early-career years can provide evidence to strengthen home-grown remote workforce development.

**Method:** This is a cross-sectional study of early-career (post-internship years 1–5) Indonesian doctors, involving an online self-administered survey on demographic characteristics, and; locations of upbringing, medical clerkship (placement during medical school), internship, and current work. Multivariate logistic regression was used to test factors associated with current work in remote districts.

**Results:** Of 3,176 doctors actively working as clinicians, 8.9% were practicing in remote districts. Compared with their non-remote counterparts, doctors working in remote districts were more likely to be male (OR 1.5,CI 1.1–2.1) or unmarried (OR 1.9,CI 1.3–3.0), have spent more than half of their childhood in a remote district (OR 19.9,CI 12.3–32.3), have completed a remote clerkship (OR 2.2,CI 1.1–4.4) or internship (OR 2.0,CI 1.3–3.0), currently participate in rural incentive programs (OR 18.6,CI 12.8–26.8) or have previously participated in these (OR 2.0,CI 1.3–3.0), be a government employee (OR 3.2,CI 2.1–4.9), or have worked rurally or remotely post-internship but prior to current position (OR 1.9,CI 1.2–3.0).

**Conclusion:** Our results indicate that building the Indonesian medical workforce in remote regions could be facilitated by investing in strategies to select medical students with a remote background, delivering more remote clerkships during the medical course, deploying more doctors in remote internships and providing financial incentives. Additional considerations include expanding government employment opportunities in rural areas to achieve a more equitable geographic distribution of doctors in Indonesia.

## Introduction

More than 90% of the population in the Asia Pacific region live in low- and middle-income countries (LMICs). Of these, nearly two-thirds reside in rural areas. Many of these countries have fewer than one doctor per 1,000 population (1). This, compounded by significant geographical maldistribution of doctors, means that the doctor-to-population ratios in some rural or remote regions is 10–75% lower than in urban areas of the same country (2–6). As doctor shortages negatively affect access to care, the World Health Organization (WHO) has recommended policies to increase health-workforce supply in rural and remote areas around four key dimensions: (1) educational, including interventions aimed at the medical training phase; (2) regulatory, including mandated rural postings and expanding the authorities of rural health workers; (3) financial incentive provisions; and (4) personal and professional supports, including strategies to provide various living amenities and facilitate professional development of the rural health workforce (7).

Studies highlight successful rural pathway initiatives in increasing rural doctor supply, that expand from medical student selection processes, offering rural medical training, and extend to providing exposure to rural clinical settings (8–13). Selection of at least 25% of medical students from a rural background and providing at least a year of rural clinical training during medical school have been successful in increasing the proportion of doctors working rurally in Australia (9, 10, 14–16). Thailand's comprehensive strategies to recruit medical students from rural regions, clinical clerkships in rural settings and provide scholarships tied to compulsory return-of-service, have jointly led to higher rural doctor retention (17, 18). China's rural-oriented tuition-waived medical education (RTME), which combines targeted recruitment of medical students from rural areas and obligatory rural service at the end of the qualification, was associated with a 12% increase in the number of rural physicians within 4 years (19).

Studies also emphasized the importance of intervention beyond medical education to recruit more doctors working rurally. The initial job upon graduation has been found to be critical in influencing work turnover among doctors in India (20). Internship, as a physician's first job, has potential to sustain the rural pathway of medical education. Doctors with an internship in non-metropolitan areas were more likely to practice in the same areas subsequently (21, 22). Compulsory or voluntary rural postings in Chile and the Philippines, mainly targeting junior doctors, also have potential to influence future practice locations of medical graduates (23).

This study focuses on Indonesia, a country with 1 doctor for every 4,300 people (24), substantially below the WHO recommendation of 1 per 1,000 (25). Eleven per cent of Indonesia's population resides in 122 government-defined remote districts (26). On average, remote districts have a doctor-to-population ratio of 1 per 6,180 population, and a doctor-to-area ratio of 1 per 170 km^2^, which contrasts with non-remote districts which are much better supplied, having average ratios of 1 doctor per 4,150 population and 1 doctor per 20 km^2^ (24). Besides the limited infrastructure and lack of health facilities, such geographically imbalanced distribution may be influenced by the decentralization systems that give district governments the authority to hire and fire health workers (27, 28).

As of 2020, Indonesia had 88 medical schools—59% of which are privately owned—producing around 10,000 graduates annually. Undergraduate medical education in Indonesia involves 3–4 years of basic medical science (mostly in a classroom setting) and 1–2 years of clerkship or clinical placements in teaching hospitals and the community. After completing medical school, doctors complete a one-year-long medical internship which involves them practicing under supervision in hospitals and primary healthcare facilities (29). This mandated year of internship was introduced in 2010 in selected districts, then rolled out nationally in 2014. Upon completion of the internship, doctors can obtain registration to practice as a general practitioner without any further training required. An additional 3–5 years of post-graduate fellowship is required to pursue other specializations. These fellowships are mostly located in teaching and teaching-affiliated hospitals in urban areas.

In Indonesia, strategies to improve the geographic distribution of doctors have been implemented since the 1980s. Two are ongoing. The first is an opt-in post-internship rural program with a financial incentive (referred to as the “rural incentive program”). The programs run for 1–2 years with the possibility of extension, and are managed by either the national government (*Nusantara Sehat*, with an average 100 places annually) or district governments (*Pegawai Tidak Tetap*/*PTT* or voluntary contractual employment, annual national number of places undocumented). *Nusantara Sehat* requires doctors to be unmarried and younger than 35 years. It provides doctors with around IDR11,000,000 (USD782) of monthly income from the national government, while the PTT doctors' monthly incomes vary from IDR4,000,000 to IDR20,000,000 (USD284-1422). These incomes are higher than the base salary for government-employed doctors of IDR2,700,000 (USD192) (30). The second strategy is the expansion of the medical internship program to include more districts for intern postings. This has resulted in 46% of interns being deployed to rural districts, and 14% to remote districts (29).

There are some early signs that geographic distribution of doctors has improved since these strategies were implemented. From 2014 to 2018, the doctor-to-population ratio in Indonesia's remote districts increased from an average of 1 per 7,060 to 1 per 6,180 population, reflecting remote-population growth of 4% and a 19% increase in the number of remotely located doctors (31, 32). However, the factors that specifically relate to better geographic distribution remain under-researched. While location of origin has been revealed as one of the reasons for doctors working in rural or remote Indonesia (33, 34), no study has explored the association between location of undergraduate education and subsequent work. Dasman et al. (35) reported that poor experience during rural internship demotivated young doctors from continuing to work in rural areas (35), yet, the study was limited to one Indonesian province.

This paper addresses the evidence gap by investigating factors associated with Indonesian doctors working in remote districts. The focus is on doctors within 5 years post-internship, as this is a period when the Indonesian government uses strategies to improve doctors' geographic distribution. In addition, location choices made at the key formative early-career stage may impact subsequent work location preferences (20, 36). Understanding these factors would inform future design of effective policies and programs.

## Materials and Methods

### Study Participants and Ethics

A cross-sectional nationwide online survey was administered to Indonesian early-career doctors who, at the time of data collection, were at post-internship years 1–5. Because surveys among physicians generally have low response rates, especially when done online (37, 38), we invited the entire cohort of medical graduates who completed their internship between 2015 and 2018 to participate (referred to hereafter as the “MoH internship population”), offering vouchers in a raffle to improve participation. The email invitations to participate in the survey were sent by the Indonesian Ministry of Health (MoH), which holds medical graduates' contact information collected at the time of internship application. The survey was anonymous, administered using Qualtrics™ and in the Indonesian language, with informed opt-in consent from all participating doctors. Ethics approval for the survey was obtained from the Monash University Human Research Ethics Committee, approval number 16922.

### Data Collection

Survey questions were drawn from national-scale medical workforce surveys in other countries, including Australia's MABEL survey (Medicine in Australia: Balancing Employment and Life) (39), the Community Service Officers Exit Survey in South Africa (40), a medical students survey in China (41) and several LMICs surveys (42, 43). These were adapted to the Indonesian context, based on a comprehensive literature review that specifically sought to hypothesize factors associated with rural practice in the Asia-Pacific LMICs context.

The survey questions, wording, and structure were extensively discussed and revised by the research team of experienced rural health workforce researchers. The team also consulted with 2 Indonesian MoH staff with experience in medical workforce and 3 Indonesian academics in health workforce policy, to inform the brevity and accuracy of the survey, relative to the research question. Two survey pilots were conducted in December 2018 to January 2019, and May 2019, with volunteer early-career Indonesian doctors. Pilot feedback was used to further refine the survey instrument, including reducing the length—from 48 to 34 questions—and rewording some questions. The final administered survey (see Supplementary Material) covered: undergraduate training and internship; location of upbringing; current and past work experiences; and demographic characteristics. The survey was online for 5 weeks between August and September 2019, and extended for 2 weeks in October 2019.

### Statistical Methods

We used summary statistics to describe respondent characteristics, and multivariate logistic regression to estimate associations between a range of factors of interests (independent variables) and the key outcome (dependent variable) which was “currently working in remote district”. Remote districts referred to those classified as underdeveloped by Presidential Regulation 131/2015, based on measures of geographic characteristics, socioeconomic status, human resources, built infrastructure, fiscal capacity, accessibility, and vulnerabilities to natural disaster (44). On average, remote districts have significantly worse doctor-to-population and doctor-to-area ratios than do non-remote districts. Remote districts are also targeted for affirmative policies, including for allocation of special funds and targeting of educational and health programs (45, 46).

**Figure 1:**
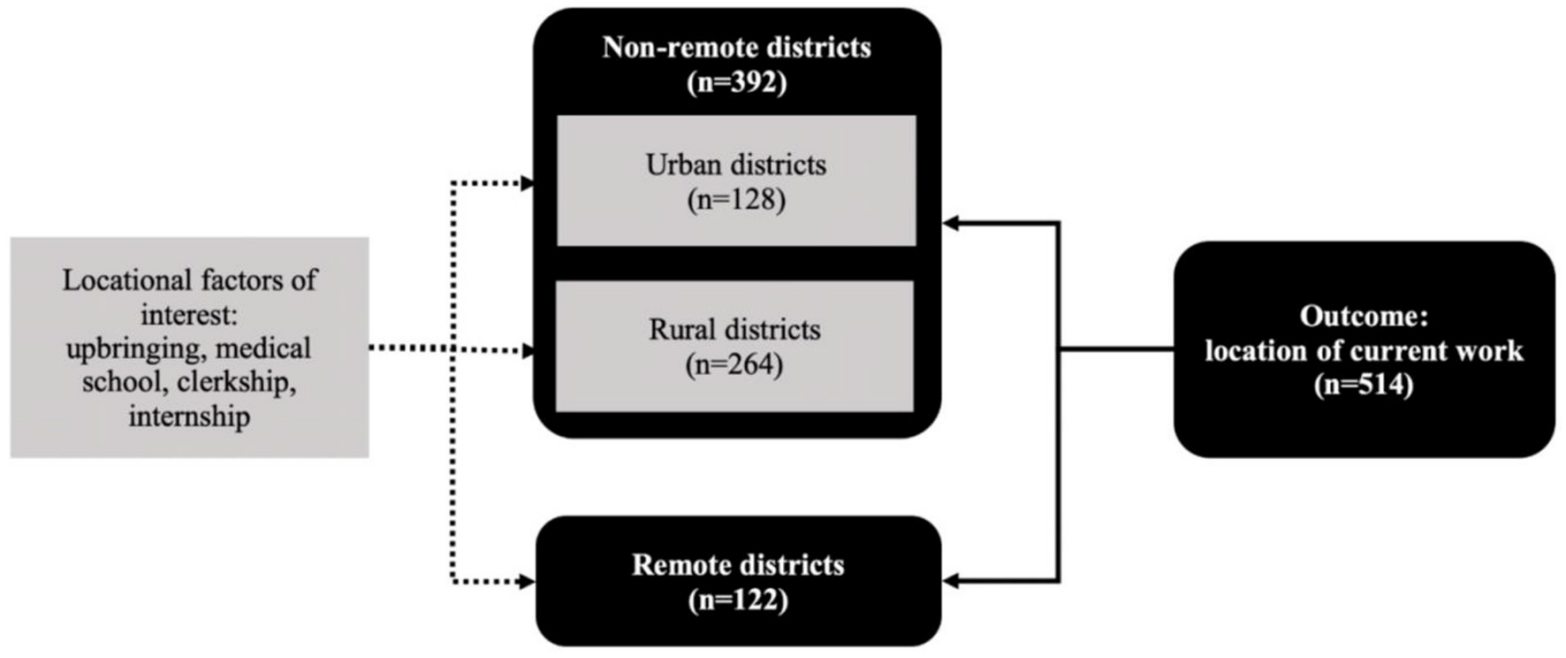
Classifications of outcome and locational factors of interest.

To further explore the effects of locational independent variables—including location(s) of the doctors' upbringing, medical school, medical clerkships and internship—non-remote districts were further classified into rural and urban. Non-remote districts were classified as rural if they had at least 50% of residents living in rural villages, while the remainder were classified as urban (47). The use of this urban-rural taxonomy is widely-applied in Indonesian studies, with rural areas more likely to have poorer health service quality and utilization (48, 49). In total, there are 122 remote districts, 264 rural districts, and 128 urban districts in Indonesia (Figure 1).

Location of upbringing was classified based on the response to the question “In what province, district, and sub-district did you live the longest up to the age of 18?”. Medical school location was based on the response to “In what medical school in Indonesia did you complete your basic medical degree?” For clerkship location, respondents were asked to list up to three sites (province and district) where their clerkships were based. These locations were coded as remote, rural or urban.

Respondents were asked whether they were participating in specific workforce programs (*Nusantara Sehat*/*PTT*/company doctor/others). Those in *Nusantara Sehat* and *PTT* were classified as “currently in a rural incentive program” and others as “currently not in a rural incentive program.” We also collected information on: previous post-internship work in any rural or remote location (*Nusantara Sehat/PTT*/Others), whether they were government employees (Yes/No), gender (Female/Male/Other), and relationship status (Unmarried/Married with children/Married with no children).

StataIC-v13 (StataCorp) was used for all statistical analyses. We performed univariate analyses to investigate associations between the outcome and each of the factors of interest. To identify potential collinearity, univariate regression was also performed between the factors of interest; those with weak association with the outcome (*p*-value > 0.05) were excluded in the multivariate model, except for age. Missing responses were categorized as “unknown” to retain them in the final modeling, which used listwise deletion. To investigate sample representativeness, data on gender, medical school type, internship location, and *Nusantara Sehat* participation of the respondents were compared with aggregate data on the MoH internship population.

## Results

### Respondent Characteristics

Of 31,510 emails sent to the MoH internship population, 17,981 were opened (57% contact rate). The email announcement and WhatsApp announcement yielded 5,199 responses downloadable via Qualtrics (29% of the contact rate), with 4,432 responses (25% of contacted respondents) from doctors who had completed their internship between 2015 and 2018 and who provided information on their internship location. The number of responses meets the minimum of required sample estimation with the known proportion of doctors working in remote districts 7.5%, total MoH internship population 31,510, 95% confidence interval and 0.01 precision (minimum sample size 2,459). Compared to the target population, survey respondents were representative by gender (61.8% female, survey respondents; 63.2% female, MoH internship population). The proportion of respondents who graduated from private medical schools (47.1%), and who completed internships in remote districts (11.6%), were slightly lower than for the MoH internship population (51.3 and 14.6%, respectively). The proportion of surveyed respondents participating in *Nusantara Sehat* (2.6%), however, was higher than that of MoH internship population (1.5%).

Of 4,432 respondents, 3,176 were working as clinicians at the time of the survey, and were included in analyses. Of these, 8.9% were working in remote districts when surveyed. Excluding unknown or missing information, 61.8% of respondents were female, 53.4% were unmarried, and the age range was 24 to 38 years (mean 27.7, SD 3.2). Doctors with a remote upbringing, remote clerkship, or remote internship, comprised 4.6, 2.5 and 11.9%, respectively of respondents (Table 1). More than half of the respondents were within 24-month of completing internship, while 21.6% mentioned that they had worked in a rural or remote location before commencing their current work.

**Table 1:** Sociodemographic characteristics of respondents.

**Characteristics**	**Groups**	**Number of respondents (%)**	**% working in remote districts**
Age (years)	27 and less	1,542 (48.6)	9.2
	28 and over	1,401 (44.1)	8.3
	Unknown/other	233 (7.3)	9.9
Gender	Female	1,791 (56.4)	6.7
	Male	1,085 (34.1)	9.2
	Unknown/other	300 (9.5)	9.6
Marital–parental status	Married–with 1+ child	878 (27.6)	4.9
	Married–without children	488 (15.4)	6.5
	Unmarried–with or without children	1,567 (49.3)	9.8
	Unknown/other	243 (7.7)	8.6
Upbringing location	Urban district^a^	2,222 (70.0)	5.6
	Rural district^b^	803 (25.3)	5.7
	Remote district^c^	145 (4.6)	58.7
	Unknown/other	6 (0.2)	0
Medical school location	Urban district^a^	3,041 (95.7)	7.8
	Rural district^b^	130 (4.1)	10.7
	Unknown/other	5 (0.2)	0
Medical school type	Private^d^	1,516 (47.7)	6.3
	Public^e^	1,655 (52.1)	9.2
	Unknown/other	5 (0.2)	0
Clerkship location^f^	Entire clerkship spent in urban districts^a^	1,557 (49.0)	8.2
	Any clerkship time in rural districts^b^	1,327 (41.8)	6.1
	Any clerkship time in remote districts^c^	78 (2.5)	27.8
	Unknown/other	204 (6.7)	8.4
Internship location^g^	Urban district^a^	1,429 (45.0)	5.4
	Rural district^b^	1,369 (43.1)	6.4
	Remote district^c^	378 (11.9)	22.5
Time since internship completion (months)	Up to 12	658 (20.7)	8.7
	13–24	1,043 (32.8)	8.4
	25–36	841 (26.5)	7.8
	37–48	558 (17.6)	6.2
	More than 48	58 (1.8)	8.3
	Unknown/other	18 (0.6)	5.0
Currently in government employment^h^	Yes	551 (17.4)	10.8
	No	2,625 (82.6)	7.3
Current participation in a rural incentive program^i^	Yes	396 (12.5)	41.2
	No	2,780 (87.5)	4.2
Previous post-internship work in rural or remote locations	None	2,355 (74.2)	5.3
	Yes, in incentive program^i^	280 (8.8)	24.7
	Yes, not in incentive program^j^	369 (11.6)	10.9
	Unknown/other	172 (5.4)	8.9

*^a^Urban districts are non-remote districts that have 50% or less of population living in rural villages, according to Head of Central Bureau of Statistics Regulation 37/2010.*

*^b^Rural districts are non-remote districts that have more than 50% of population living in rural villages, according to Head of Central Bureau of Statistics Regulation 37/2010.*

*^c^Remote districts are those defined as isolated, border or island districts according to Presidential Regulation 131/2015.*

*^d^Private medical schools are those funded by a private or non-government organization.*

*^e^Public medical schools are those funded by the government.*

*^f^Clerkship or clinical rotation is a phase in the undergraduate medical course, usually in the final year(s) of study, in which students are under supervision and do not have full authority to treat patients. In Indonesia, clerkships take 1.5–2 years. During the clerkship, medical students are placed in teaching hospitals or affiliation hospitals, in accordance with their medical school's regulations. For example, one medical school may allocate the entire clerkship to one hospital's pediatrics department, while another may distribute the clerkship across more than one hospital.*

*^g^In Indonesia, internship completion is required for medical graduates to obtain registration as a doctor. Interns have full authority to treat patients.*

*^h^Government employment of doctors with a long-term (lifetime) contract, whether during candidature or at the official stage (Calon Pegawai Negeri Sipil [CPNS] or Pegawai Negeri Sipil [PNS]).*

*^i^Rural incentive programs include Nusantara Sehat and PTT. Incentive amounts may vary.*

*^j^Refers to any work experience in rural or remote locations outside the Nusantara Sehat and PTT programs. The participants may or may not have received additional financial incentives*.

### Predictors of Remote Work Location

Of those who grew up in remote districts (*n* = 145), 58.7% were practicing in remote districts, compared to 5.6 and 5.7% of those growing up in urban and rural districts, respectively. Out of 378 doctors undertaken a remote internship, 22.5% were working in a remote district when surveyed (Table 1).

Multivariate logistic regression showed that doctors working in a remote district were more likely to: be male (OR 1.5, CI 1.1–2.1); be unmarried (OR 1.9, CI 1.3–3.0); have grown up in a remote district (OR 19.9, CI 12.3–32.3); have a clerkship in a remote district (OR 2.2, CI 1.1–4.4); have undertaken an internship in a remote district (OR 2.0, CI 1.3–3.0); be enrolled in the rural incentive program when surveyed (OR 18.6, CI 12.8–26.8); and be a government employee (OR 3.2, CI 2.1–4.9). Strong associations were also found between previous post-internship work in any rural or remote district (OR 1.9, CI 1.2–3.0) and past participation in a rural incentive program (OR 2.0, CI 1.3–3.0) with current work in remote districts. Univariate analyses showed no association between working in a remote district and age, years of post-internship, or medical school location. The odds of working in a remote district were similar for married doctors with children and those without children. An association between attending a public medical school and remote work was evident in the univariate model but was not significant in the multivariate model (Table 2).

**Table 2:** Odds ratio of working in remote districts (*n* = 3,176).

	**Respondent characteristic**	**Univariate logistic regressions**		**Multivariate logistic regressions**	
		**OR-crude**	**95% CI**	**OR-adjusted**	**95% CI**
1	28 years-old and over	0.89	0.69, 1.15	0.92	0.66, 1.30
2	Male	1.47*	1.13, 1.90	1.51*	1.09, 2.10
3	Marital status (Reference married with 1+ child)				
	Married without children	1.41	0.90, 2.21	1.16	0.66, 2.04
	Unmarried–with or without child	2.21**	1.58, 3.08	1.94*	1.27, 2.97
4	Upbringing location (Reference urban district)				
	Rural district	0.96	0.69, 1.34	0.87	0.59, 1.29
	Remote district	24.87**	17.05, 36.28	19.94**	12.32, 32.28
5	Medical school located in rural district	1.47	0.86, 2.52	Excluded	
6	Public medical school	1.68**	1.30, 2.17	1.32	0.96, 1.82
7	Clerkship location (Reference entire clerkship in urban district^1^)				
	Any clerkship time in rural district	0.72**	0.55, 0.95	0.84	0.58, 1.20
	Any clerkship time in remote district	4.63**	2.79, 7.67	2.17*	1.07, 4.40
8	Internship location (Reference urban district)				
	Rural district	1.11	0.82, 1.49	0.84	0.58, 1.20
	Remote district	4.87**	3.55, 6.67	1.96*	1.29, 2.96
9	Time since completing internships (Reference up to 12 months)				
	13–24 months	1.01	0.73, 1.42	Excluded	
	25–36 months	0.93	0.65, 1.33		
	37–48 months	0.80	0.53, 1.20		
	More than 48 months	1.13	0.47, 2.74		
10	Currently in government employment	1.39*	1.03, 1.88	3.23**	2.14, 4.87
11	Current participation in a rural incentive program	26.40**	18.90, 36.91	18.56**	12.84, 26.83
12	Previous post-internship work in rural or remote locations (Reference no rural or remote post-internship work)				
	In rural incentive program	5.66**	4.13, 7.75	1.99*	1.32, 3.01
	Not in rural incentive program	2.20**	1.55, 3.13	1.90*	1.22, 2.96

*p-value ≤ 0.05, **p-value < 0.001.

*The unknown category was included in the analysis; no strong association found hence these are not shown*.

## Discussion

To our knowledge, this is the first national quantitative study exploring factors associated with doctors' work locations in remote districts in Indonesia. Nine percent of early-career doctors surveyed (up to 5 years post-internship) were working in remote districts. This compares to 11% of Indonesia's population who live in these areas (26). Critically, our study identified factors strongly associated with working in remote districts. These include growing up in a remote district; undertaking a remote clerkship during medical school; undertaking a remote internship; working as a government employee; participation in a rural incentives program; being male; unmarried; and previously having worked in any rural or remote area. The first three of these listed factors are all rural pathway factors, suggesting that workforce strategies which select students into medical school from remote areas, train them in those rural and remote locations and then employ them in rural and remote locations immediately upon graduation are key to Indonesia's future remote medical workforce.

Strong relationships between doctors' intentions or actual work in rural areas and rural background, location of secondary schooling, and having a spouse or family living in a rural area have been widely recognized by many previous LMIC studies in the Asia Pacific (3, 4, 41–43, 50–59). Importantly, this research is the first quantitative evidence confirming such strong associations with Indonesian doctors' actual work locations. Our study is also the first to show that, of all the factors of interest, a remote upbringing has the strongest association with remote practice—increasing the odds of remote practice by a substantial 20 times. In contrast, there was no difference in the odds of remote work between doctors who grew up in urban and those who grew up in rural areas. This suggests that exposure to remote places during training, and perhaps to specific remote regions with which doctors may already have a connection, may be effective strategies to encourage remote work choices (60, 61). According to our findings, remote students are proportionally underrepresented in Indonesian medical schools, comprising <5% of students. Without intervention, it is possible that the already low proportion of students who come from remote areas could reduce over time, as has happened, for example, in the United States (62). Given these circumstances, policies and programs that support recruiting undergraduate medical students from remote districts, specifically, are recommended.

Doctors who participated in remote clerkships as medical students had twice the odds of working in a remote district compared to those who only had urban clerkships. This finding indicates that existing evidence, drawn from other countries, of associations between rural clinical placements and rural work preference is relevant for Indonesia (10, 14, 63, 64). This is the case even though Indonesian medical students are assigned by their medical school to rural and remote clerkships; students in many other countries can self-select into these clerkships (64, 65). Given this evidence, increasing the number of remote hospitals and health services which are affiliated with Indonesian medical schools and expanding the number of remote clerkship training weeks during medical school may further improve remote medical workforce outcomes.

Our findings are consistent with other evidence of a positive association between having completed rural internships and subsequent rural work (21, 66, 67). In Indonesia, there are a limited number of internship positions in urban-located hospitals. This forces some interns to choose between doing rural or remote internships or delaying their internship in the hope of getting an urban internship in a subsequent round of internship allocations. The financial assistance for interns' salary provided by the MoH is higher in remote posts, and non-financial support such as, supervisor training and program standardization are also provided (68). This program needs to be expanded to increase the number of doctors working in Indonesian rural locations. Other countries may consider investing in rural internship program as a part of the rural pathway to strengthen efforts to build rural medical workforce.

We found that the opt-in *Nusantara Sehat* and *PTT* incentive programs are positively associated with current work in remote Indonesia, consistent with earlier studies demonstrating that opt-in rural incentive programs help address rural doctor shortages (23, 69). We also found that doctors who had ever participated in those programs were more likely to be currently working in remote districts. This suggests that the rural experiences gained through the *Nusantara Sehat* and *PTT* programs could be an important part of a pathway to rural practice. This extends beyond the internship year, since these programs are associated with subsequent rural work even after the program incentives are no longer being received.

The strong association that we identified between being a government employee and practicing in remote districts is interesting. Although the positions for government employment are equally available in remote and non-remote districts, the competition may be tougher in the non-remote districts. Further, working in remote areas as a government employee may provide an additional advantage for priority access to scholarships for non-general practice training, for early career doctors wanting to be a specialist. Other evidence has shown that the opportunity for government employment is an incentive for doctors to work in remote locations (70). Indonesian government employees are more likely to be eligible for continuing education scholarships, which are very attractive to medical graduates (71). Also, government employees are permitted to earn additional income from second or third jobs, which is similarly attractive (58, 72, 73). These findings suggest that the longer-term success of stand-alone strategies such as rural incentive programs at the early-career stage could be consolidated by simultaneously increasing opportunities for government appointments in the hardest-to-staff remote locations, or by expanding private job opportunities in remote areas. Concern that government employees with second or third jobs may provide a lower quality of service in government health facilities needs to be managed (74, 75).

Our findings indicate that district governments could have a greater role in developing a home-grown medical workforce—an important highlight for a decentralized nation like Indonesia. Since a remote upbringing has the strongest association with remote work for early-career doctors in Indonesia, remote-district governments could facilitate the entry of local students to medical school, by establishing collaborations with the schools, or providing bonded scholarships. Such collaborations could extend to nominating their district hospitals as sites for clerkships and internships. Remote-district governments could also prioritize government employment for doctors. However, support from the national government should be continued, especially in attracting more doctors to rural and remote locations with its rural financial incentive program and the nationwide internship program.

This study is exploratory and could be extended through ongoing research. First, further research could explore aspects of rural “place” classifications at a more nuanced level, rather than the binary outcome (remote/non-remote) used in this study. Second, the association between duration or design of any rurally-enhanced program and doctors' subsequent work location should be explored in more detail. This includes considering whether a longer duration of clerkship or internship experience, or whether longer exposures in community settings, are more strongly associated with remote or rural workforce outcomes, as has been shown in other countries (76, 77). Third, exploring elements of the internship program that may be more strongly associated with remote practice after the internship would provide more information for future program improvement. Fourth, understanding the long-term retention of doctors in remote areas beyond the internship or rural financial incentive program is essential to identify further efforts required to increase sustainability of the remote medical workforce. It is possible that for retention, working conditions and ongoing training or up-skilling opportunities become imperative (78).

We acknowledge some limitations in this study. This was a self-administered survey with retrospective recollection of details about past characteristics including geographic locations, hence, self-selection and recall bias may occur. As the invitation to the survey was announced through email and online platforms, clinicians not regularly using them may have been under-represented amongst respondents. As described in the Results section, several characteristics of the doctor population were assessed for representativeness. Respondents participating in *Nusantara Sehat*, one of the rural incentive programs, were overrepresented, which may have led to overestimation of its association with remote practice. However, the odds ratio was high (18.9) with a small *p*-value (*p* < 0.001); thus, a type-I error is unlikely in this case. Public medical school graduates were also overrepresented. However, in multivariate analysis this characteristic showed no association with remote practice, and thus did not affect interpretation of the results. Finally, this study did not adjust for other predictors of rural preference found in other studies such as job-related factors (i.e., quality of relationships with colleagues, access to specialist consultations, health facility infrastructure and equipment) (79–81), and locational factors (i.e., access to transportation, socioeconomic development, population density, and health insurance coverage) (82–85).

## Conclusion

Our study identifies strong associations between working in remote districts and multiple factors related to rural training pathways (selection, rural training and exposure, professional support, and type of employment). These results indicate that building the Indonesian medical workforce in remote regions could be facilitated by investing in strategies to select medical students with a remote background, delivering more remote clerkships during training, employing more doctors in remote internships, and providing financial incentives for remote work. This would require establishing a more extensive network of remote clerkships for medical students by broadening medical school networks with affiliated-teaching hospitals and community practices. Additional policies include expanding highly sought-after government employment opportunities in rural and remote areas. These strategies are strongly tied to the issue of developing a connected rural pathway to “grow your own,” consistent with the 2010 WHO global policy recommendations about increasing access to health workers.

## Data Availability Statement

The data presented in this article are not readily available because they are subject to the requirements of the Monash University Human Research Ethics Committee that they cannot be shared publicly. Requests to access the datasets should be directed to Likke Prawidya Putri: likke.putri@ugm.ac.id or likkepp@gmail.com.

## Ethics Statement

This study was reviewed and approved by the Monash University Human Research Ethics Committee. Informed opt-in consent was given by all participants in accordance with the requirements of the study's ethics approval.

## Author Contributions

LP, DR, BO'S, and RK designed the study. LP analyzed the data and wrote up the initial draft. DR, BO'S, and RK guided the analysis and interpretation of the results, and drafting of the paper. RK was the principal supervisor overseeing the study. All authors contributed to the article and approved the submitted version.

## Conflict of Interest

The authors declare that the research was conducted in the absence of any commercial or financial relationships that could be construed as a potential conflict of interest. The handling editor is currently organizing a Research Topic with one of the authors BO'S.
