# Supplementary material for: Factors Associated With Working in Remote Indonesia: A National Cross-Sectional Study of Early-Career Doctors
Source: Front Med (Lausanne). 2021 May 13;8:594695. doi: 10.3389/fmed.2021.594695 (PMC8155628; doi:10.3389/fmed.2021.594695)
Supplement: Supplementary file 1 [file Table_1.DOCX]

Appendix 1. Final survey questions: paper version

| **BLOCK A: Location of upbringing** | |
| --- | --- |
| A01 | In what province, district, and sub-district did you live the longest up to the age of 18 years? |
|  | Province: (dropdown list) |
|  | District: dropdown list) |
|  | Sub-district: (dropdown list) |
|  | **AND** |
|  | Name of village: (*Please fill with ‘99’ if you don’t remember, don’t know or not willing to answer)* |
|  | |
| A02 | **Number of years spent in this location:** |
|  | (0 – 18 years) |
|  | |
| A03 | Up to the age of 18 years, how many years in total (if any) did of live in rural or remote areas? |
|  | (0 – 18 years) |
|  | **SKIP to B01 for those answering ‘0’** |
|  |  |
| A04 | Where is the rural or remote area where you lived for the most time up to the age of 18 years?  *Please fill with ‘99’ if you don’t remember, don’t know or not willing to answer* |
|  | Province: (dropdown list) |
|  | District: (dropdown list) |
|  | Subdistrict: *Please fill with ‘99’ if you don’t remember, don’t know or not willing to answer)* |
|  | Village: *Please fill with ‘99’ if you don’t remember, don’t know or not willing to answer)* |

| **BLOCK B: Undergraduate Training and Internship** | | | | | | | | | |
| --- | --- | --- | --- | --- | --- | --- | --- | --- | --- |
| B01 | In what month and year did you complete your basic medical degree? | | | | | | | | |
|  | Month (dropdown list) | | Year (dropdown list) | | | | | | |
|  | | | | | | | | | |
| B02 | In what medical school in Indonesia did you complete your basic medical degree? | | | | | | | | |
|  | UNIVERSITAS AIRLANGGA | | | | UNIVERSITAS INDONESIA | | | | |
|  | UNIVERSITAS ANDALAS | | | | UNIVERSITAS LAMPUNG | | | | |
|  | UNIVERSITAS ATMAJAYA | | | | UNIVERSITAS PADJADJARAN | | | | |
|  | UNIVERSITAS BRAWIJAYA | | | | UNIVERSITAS SEBELAS MARET | | | | |
|  | UNIVERSITAS DIPONEGORO | | | | UNIVERSITAS SRIWIJAYA | | | | |
|  | UNIVERSITAS GADJAH MADA | | | | UNIVERSITAS UDAYANA | | | | |
|  | UNIVERSITAS HASANUDDIN | | | | OTHERS: ______ | | | | |
|  |  | | | | | | | | |
| B03 | Please mention in which locations (name of the district) you did your clinical rotation (both hospital and community) and how long the duration is: | | | | | | | | |
|  | Province: | District: | | Total time spent in location:   - Less than 3 months - From 3 months to less than 1 year - More than 1 year - N/A | | | | | |
|  | (Up to 3 answers) |  | |  | | | | | |
|  | | | | | | | | | |
| B04 | In which month and year did you complete your internship program? | | | | | | | | |
|  | Month (dropdown list) | | Year (dropdown list) | | | | | | |
|  | | | | | | | | | |
| B05 | In what province, district, and facility did you complete your internship program? | | | | | | | | |
|  | Province: (dropdown list) | | | | | | | | |
|  | District: dropdown list) | | | | | | | | |
|  | Hospital (dropdown list) | | | | | | | | |
|  | Primary healthcare facility  (fill out form, *Please fill with ‘99’ or name of sub-district if you don’t remember, don’t know or not willing to answer*) | | | | | | | | |
|  |  | | | | | | | | |
| B06 | What is (are) your reason(s) of taking the internship in this location? | | | | | | | | |
|  | *Tick all that apply* | | | | | | | |  |
|  | 1. The quota in the location I desired was not available | | | | | | | |  |
|  | 1. Near to hometown and/or family and/or partner and/or partner’s family | | | | | | | |  |
|  | 1. Good career prospect in the long-term | | | | | | | |  |
|  | 1. Prior knowledge that this place offers better opportunity to earn extra income on top of MoH allowance | | | | | | | |  |
|  | 1. Prior knowledge that accommodation will be provided | | | | | | | |  |
|  | 1. Good opportunity and/or support to continuing education | | | | | | | |  |
|  | 1. Good infrastructure / transportation access in the location | | | | | | | |  |
|  | 1. Better opportunity for professional development and improving clinical competence | | | | | | | |  |
|  | 1. Adequate schooling choices for children | | | | | | | |  |
|  | 1. Good employment opportunity for partner | | | | | | | |  |
|  | 1. OTHERS: | | | | | | | |  |
|  |  | | | | | | | | |
| B07 | During my internship program: | | | | | | | | |
|  |  | | | | | Agree | Disagree | N/A | |
| 1. The additional income I earned (from local government and/or health facilities) was fair | | | | | |  |  |  | |
| 1. The health facility had adequate medical equipment and drugs | | | | | |  |  |  | |
| 1. I experienced significant professional development | | | | | |  |  |  | |
| 1. I experience significant development in clinical competence | | | | | |  |  |  | |
| 1. I was satisfied with the level of professional responsibility entrusted to me | | | | | |  |  |  | |
| 1. I was satisfied with the level of collaboration with other health professionals at the facility | | | | | |  |  |  | |
| 1. I was satisfied with the support (training or coaching) from my supervisor(s) | | | | | |  |  |  | |
| 1. I feel that I significantly contributed to the health of the community I served | | | | | |  |  |  | |

| **BLOCK C. Postgraduate Fellowship /Specialization Training** | | |
| --- | --- | --- |
| C01 | Are you currently enrolled in a specialist education or fellowship program?  *Notes: currently enrolled means that you are already accepted or have not officially graduated* | |
|  | - Yes, in: _________ |  |
|  | - No |  |

| **BLOCK D. Current work** | | | | | | |
| --- | --- | --- | --- | --- | --- | --- |
|  | This section will ask about your current work | | | | | |
|  | | | | | | |
| D01 | Are you currently a government employee (PNS) or the candidate of PNS (CPNS)? | | | | | |
|  | - Yes | | | | | |
|  | - No | | | | | |
|  | | | | | | |
| D02 | *[This question will NOT appear for those currently in specialization / fellowship program]*  Are you currently a medical specialist? | | | | | |
|  | - Yes, in: _________ |  | | | | |
|  | - No |  | | | | |
|  | | | | | | |
| D03 | *[This question will NOT appear for those currently in specialization / fellowship program]*  Are you currently working clinically in Indonesia?  *Notes: Clinical work defined as any activity involving diagnosing and/or treating patients* | | | | | |
|  | - Yes |  | | | | |
|  | - No | **Skip to D09** | | | | |
|  | | | | | | |
| D04 | **The next 3 questions will discuss the location (province, district, sub-district, village) where you are currently working.**  Where is the location of your current work?  *Notes:*  *Place of work: if you are working in more than 1 facility in different sub-district, then fill with your main place of work*  *If you are in a residency program, please fill with your current placement* | | | | | |
|  | Province: | | | | | |
|  | District: | | | | | |
|  | **AND** | | | | | |
|  | Subdistrict: | | | | | |
|  | Village:  *Please fill with ‘99’ if you don’t remember, don’t know or not willing to answer* | | | | | |
|  | Name or address of the facility  *Please fill with ‘99’ if you don’t remember, don’t know or not willing to answer* | | | | | |
|  | | | | | | |
| D05 | How long have you been working in this location (this village or address mentioned above)? | | | | | |
|  | ______ years | | | | | |
|  | | | | | | |
| D06 | *[This question will NOT appear for those currently in specialization / fellowship program]*  Are you working here under the special assignment? | | | | | |
|  | - Yes, I am working here under the *Nusantara Sehat* program | | | |  | |
|  | - Yes, I am working here under the PTT program | | | |  | |
|  | - Yes, I am working here under the WKDS program | | | |  | |
|  | - Yes, I am working here as a rotating doctor | | | |  | |
|  | - Yes, I am working here on: _________ | | | |  | |
|  | - None of the above | | | |  | |
|  | | | | | | |
| D07a | What is (are) your reason(s) of working in this location? | | | | | |
|  | 1. Near to hometown and/or family and/or partner and/or partner’s family | | | | |  |
|  | 1. Good career prospects for the long term | | | | |  |
|  | 1. Better opportunity to earn high income | | | | |  |
|  | 1. Good opportunity and/or support to continuing education | | | | |  |
|  | 1. Good infrastructure / transportation access in the location | | | | |  |
|  | 1. Good working environment for professional development and improving clinical competence | | | | |  |
|  | 1. Adequate schooling choices for children | | | | |  |
|  | 1. Good employment opportunity for partner | | | | |  |
|  | 1. The health facilities have an adequate medical equipment and drugs | | | | |  |
|  | 1. As return-for-service | | | | |  |
|  | 1. OTHERS: | | | | |  |
|  | | | | | | |
| D07b | From all of these factors, which one is the most important for you to work in this location? | | | | | |
|  |  | | | |  | |
|  | 1. Near to hometown and/or family and/or partner and/or partner’s family | | | |  | |
|  | 1. Good career prospect for in long-term | | | |  |  |
|  | 1. Better opportunity to earn high income | | | |  |  |
|  | 1. Good opportunity and/or support to continuing education | | | |  |  |
|  | 1. Good infrastructure / transportation access in the location | | | |  |  |
|  | 1. Good working environment for professional development and improving clinical competence | | | |  |  |
|  | 1. Adequate schooling choices for children | | | |  |  |
|  | 1. Good employment opportunity for partner | | | |  |  |
|  | 1. The health facilities have an adequate medical equipment and drugs | | | |  |  |
|  | 1. As return-for-service | | | |  |  |
|  | 1. OTHERS: | | | |  | |
|  | | | | | | |
| D08a | In a typical working week, how many hours do you work in total? | | | | | |
|  | ___ hours | | | | | |
|  | | | | | | |
| D08b | In a typical working week, how many hours do you work in these locations / facilities? | | | | | |
|  | - In hospital, government-owned | | ___ hours | | | |
|  | - In hospital, private-owned | | ___ hours | | | |
|  | - In specialist clinic, private-owned | | ___ hours | | | |
|  | - In primary care clinic, private-owned | | ___ hours | | | |
|  | - In Puskesmas | | ___ hours | | | |
|  | - In maternity clinic | | ___ hours | | | |
|  | - In other facility: _________ | | ___ hours | | | |
|  | | | | | | |
| D09 | Which of the following statements describe your current situation?  (Tick all that apply) | | | | | |
|  | - Doing medical-related but non-clinical work (e.g. teaching, research, in pharmaceutical company, in BPJS Kesehatan, committee work) | | |  | | |
|  | - Holding managerial position in a health facility (e.g. director or manager or structural position in the hospital, head of primary healthcare center) | | |  |  |  |
|  | - Permanent retirement from all type of paid work (i.e. not intended to continue to any paid work in the future) | | |  |  |  |
|  | - On leave (such as: sick leave, unpaid leave, maternity leave) | | |  |  |  |
|  | - OTHERS: | | |  |  |  |
|  | | | | | | |
| D10 | Where is the location where you are working / studying currently? | | | | | |
|  | Province: (dropdown list) | | |  | | |
|  | District: dropdown list) | | |  | | |
|  | Sub-district OR institution:  *Please fill with ‘99’ if you don’t remember, don’t know or not willing to answer* | | |  | | |

|  | **Past Work Experience in Rural or Remote Locations** | | |
| --- | --- | --- | --- |
| E01 | In the period after finishing internship and before starting your current job, did you work in any rural or remote location?  *Tick all that apply* | | |
|  | - Yes, I had been working under Nusantara Sehat program | | |
|  | - Yes, I had been working under PTT program | | |
|  | - Yes, I had been working under WKDS program | | |
|  | - Yes, I had been working as a rotating doctor / company doctor | | |
|  | - Yes, I had been working in rural or remote location but not under the aforementioned four programs | | |
|  | - No | | **SKIP to F01** |
|  | | | |
| E02 | Where is the location (of rural or remote location) you served?  *If you work in more than 1 rural or remote location, please fill in with the location you were posted the longer Please fill with ‘99’ if you don’t remember, don’t know or not willing to answer* | | |
|  | Province: (dropdown list) | | |
|  | District: (dropdown list) | | |
|  | Sub-district OR institution:  *Please fill with ‘99’ if you don’t remember, don’t know or not willing to answer* | | |
|  | | | |
| E03 | | How long did you work in for the aforementioned program or the rural or remote location? | |
|  | | ______ years | |

|  | **Demographic information** | | | | | | |
| --- | --- | --- | --- | --- | --- | --- | --- |
| F01 | What is your current age in years? | | | | | | |
|  | ______ years | | | | | | |
|  | | | | | | | |
| F02 | What is your gender? | | | | | | |
|  | Male | | | | | | |
|  | Female | | | | | | |
|  | | | | | | | |
| F03 | What ethnic group do you identify with? | | | | | | |
|  | Aceh | Batak | Dayak | | Minangkabau |  | |
|  | Bali | Betawi | Jawa | | Sasak |  | |
|  | Banjar | Bugis | Madura | | Sunda |  | |
|  | Banten | Chinese | Melayu | | OTHERS: |  | |
|  |  | | | | | | |
| F04 | Besides bahasa Indonesia, what **local language(s)** are you proficient in? That is, you can at least understand the meaning of a complex sentence? | | | | | | |
|  | NONE | Batak | Madura | | Musi | | OTHERS: |
|  | Aceh | Betawi | Melayu | | Nias | |  |
|  | Bali | Bugis | Minang | | Sunda | |  |
|  | Banjar | Javanese | Makassar | | Toraja | |  |
|  |  | | | | | | |
| F05 | What is your religious belief? | | | | | | |
|  | - Islam | | | - Buddhism | | | |
|  | - Christian | | | - Kong Hu Cu | | | |
|  | - Catholic - Hinduism | | | - OTHERS: | | | |
|  |  | | | | | | |
| F06 | What is your current relationship status? | | | | | | |
|  | - Married | | | | | | |
|  | - In a relationship – living together | | | | | | |
|  | - In a relationship – not living together | | | | | | |
|  | - Single | | | | | | |
|  |  | | | | | | |
| F07 | How many children do you have? | | | | | | |
|  | - 0 | | | - 2 | | | |
|  | - 1 | | | - 3 or more | | | |
